# Genome-Wide Open Chromatin Methylome Profiles in Colorectal Cancer

**DOI:** 10.3390/biom10050719

**Published:** 2020-05-05

**Authors:** Muhiddin Ishak, Rashidah Baharudin, Isa Mohamed Rose, Ismail Sagap, Luqman Mazlan, Zairul Azwan Mohd Azman, Nadiah Abu, Rahman Jamal, Learn-Han Lee, Nurul Syakima Ab Mutalib

**Affiliations:** 1 UKM Medical Molecular Biology Institute (UMBI), Universiti Kebangsaan Malaysia, Kuala Lumpur 56000, Malaysia; muhiddin@ppukm.ukm.edu.my (M.I.); ieda_baharudin@yahoo.com (R.B.); nadiah.abu@ppukm.ukm.edu.my (N.A.); rahmanj@ppukm.ukm.edu.my (R.J.); 2 Department of Pathology, Faculty of Medicine, Universiti Kebangsaan Malaysia, Jalan Yaacob Latif, Cheras, Kuala Lumpur 56000, Malaysia; isa@ppukm.ukm.edu.my; 3 Department of Surgery, Faculty of Medicine, Universiti Kebangsaan Malaysia, Jalan Yaacob Latif, Cheras, Kuala Lumpur 56000, Malaysia; ismailsagap@ppukm.ukm.edu.my (I.S.); luqman@ppukm.ukm.edu.my (L.M.); zairulazwan@ppukm.ukm.edu.my (Z.A.M.A.); 4 Novel Bacteria and Drug Discovery Research Group, Microbiome and Bioresource Research Strength, Jeffrey Cheah School of Medicine and Health Sciences, Monash University Malaysia, Subang Jaya 47500, Selangor, Malaysia

**Keywords:** colorectal cancer, open chromatin, DNA methylation, Infinium MethylationEPIC, epigenetics aberrations

## Abstract

The methylome of open chromatins was investigated in colorectal cancer (CRC) to explore cancer-specific methylation and potential biomarkers. Epigenome-wide methylome of open chromatins was studied in colorectal cancer tissues using the Infinium DNA MethylationEPIC assay. Differentially methylated regions were identified using the ChAMP Bioconductor. Our stringent analysis led to the discovery of 2187 significant differentially methylated open chromatins in CRCs. More hypomethylated probes were observed and the trend was similar across all chromosomes. The majority of hyper- and hypomethylated probes in open chromatin were in chromosome 1. Our unsupervised hierarchical clustering analysis showed that 40 significant differentially methylated open chromatins were able to segregate CRC from normal colonic tissues. Receiver operating characteristic analyses from the top 40 probes revealed several significant, highly discriminative, specific and sensitive probes such as *OPLAH* cg26256223, *EYA4* cg01328892, and *CCNA1* cg11513637, among others. *OPLAH* cg26256223 hypermethylation is associated with reduced gene expression in the CRC. This study reports many open chromatin loci with novel differential methylation statuses, some of which with the potential as candidate markers for diagnostic purposes.

## 1. Introduction

Cancer is a continuous global burden with an estimation of over 18.1 million new incidences and projected to increase in the next decade [1]. Within these statistics, colorectal cancer (CRC) contributes around 1.1 million (6%) of total cases and is ranked as the fourth most common cancer in the world [1]. CRC can occur through the accumulation of multiple genetics and epigenetics changes. Research has shown that somatic mutation in *APC*, *BRAF*, *KRAS*, *PIK3CA* and *TP53* [2,3] have been frequently observed in CRC and are considered the drivers of CRC formation. Despite many kinds of research on molecular alterations involved in CRC pathogenesis, the existing knowledge remains inadequate for early diagnosis and prognosis assessment. The CRC incidence continues to increase in recent years. In addition, there is a shift in its incidence towards younger patients who are diagnosed with advanced forms of cancer [4]. Furthermore, the cost associated with CRC management increase with the increasing stage of the disease [5] and will pose a great burden to the health sector. Further understanding of the epigenetic components in the processes involved in CRC carcinogenesis is highly desired and will unravel new biomarkers which can be utilized for diagnosis, prognosis and treatment to prevent CRC-related mortality. One of the candidates for the biomarkers could be discovered from analysing the epigenome of the tumours.

Epigenetics mechanism can be classified into DNA methylation and histone modification [6], with the former being the most widely studied mechanism. Our group has performed DNA methylation profiling on 55 paired CRCs and adjacent normal epithelial cells using Illumina HumanMethylation27k [7]. This array covers 27,758 CpG dinucleotides spanning 14,495 genes [8]. However, this array only provides information on a small part of the entire genome, mostly promoters, and could not comprehensively address the complexity of the epigenetic regulation of this disease. The Illumina Infinium HumanMethylation450K BeadChip, which is the upgraded version, provides coverage of 485,000 sites, including 99% of RefSeq genes, CpG islands, the island shores and the flanking regions, gene promoter, 5’ UTR, first exon, gene body and 3’ UTR [9]. We have also used this platform to identify differentially methylated regions in recurrent CRCs [10] and further provided the evidence of five potentially biologically important genes in recurrent CRCs that could possibly serve as new potential therapeutic targets for patients with chemoresistance, which includes *CCNEI*, *CCNDBP1*, *PON3*, *DDX43*, and *CHL1*.

Promoters are the most widely investigated region in DNA methylation studies; however other regions in the genome are also susceptible to epigenetic regulation including the microRNAs, enhancers, as well as the chromatins. Chromatin states can be broadly classified into two types: active (or open) and inactive (or compact) [11]. Generally, gene transcription occurs in regions where the chromatin conformation is more open, whereas transcriptionally silenced genes are found in regions with compact chromatin [11]. This process is assisted by the transcription factors (TFs) that bind to DNA in a sequence-specific manner to remove or move the nucleosomes to form open chromatin [12].

As ongoing epigenome studies revealed the involvement of open chromatin regions in regulation of gene expression, there is a demand for a more comprehensive tool for methylation profiling, preferably based on microarray technology. Hence, at the end of 2015, the Infinium MethylationEPIC was launched and it provides coverage of more than 850,000 regions of interest. Specifically, in addition to the >90% of contents covered in HumanMethylation450K, this array covers >200,000 ENCODE open chromatins [13]. A technical evaluation by Pidsley et al. [13] concluded that the latest array is a significant improvement over the former version. The inclusion of open chromatins can provide a new perspective and unravel new epigenetics landscape of CRC.

To date, the closest example of Infinium MethylationEPIC application in CRC is portrayed by Gallardo-Gómez et al. [14], whereby a pooled serum circulating cell-free DNA (cfDNA) from 20 individuals with no colorectal findings, advanced adenomas and CRCs was subjected to microarray profiling using this beadchip. However, the findings reported are exploratory in nature and focused on the technical performance of the platform, rather than discussing the biological findings. The authors also clarified that this limitation was due to the limited amount of cfDNA. Besides, in the same year, another research also applied ~850,000 CpG sites was conducted on six normal and 16 colon tumours. This research aimed to identify the epigenetic changes in tumour phenotypes that lead to phenotypic intratumoral heterogeneity [15]. In 2019, Gu et al. [16] profiled the DNA methylation profiles in 12 CRCs with adjacent normal colons but did not harness the information of open chromatins methylome. More recently, Wang and his colleagues have performed epigenome-wide association study (EWAS) on 334 normal colon mucosa using Infinium MethylationEPIC to estimate the normal colon biological tissue age in individuals within the three CRC risk group (low, medium and high) based on their personal adenoma or cancer history [17]. In addition, to the best of our knowledge, there have been extremely limited studies which investigate global DNA methylation profile using this beadchip which emphasize on the open chromatins area in CRC. Therefore, we aim to provide the readers with a comprehensive dataset of DNA methylation of open chromatins in CRC.

## 2. Materials and Methods

### 2.1. Clinical Specimens

The specimens from 51 CRC patients diagnosed at the UKM Medical Centre (UKMMC) were retrieved from the UMBI-UKMMC Biobank (23 pairs of tumour-adjacent normal fresh frozen colon tissues and 28 CRC tissues). The specimens were collected according to the procedures approved by the UKM Research Ethics Committee and all patients gave informed consent for their specimens to be stored and used for future research. The tissues were dissected, snap-frozen and stored in liquid nitrogen. All samples were cryosectioned and stained with haematoxylin and eosin and the percentage of tumour cells and normal cells contents were assessed by a pathologist. Only tumour samples with at least 80% cancerous cells and normal adjacent colon tissues with less than 20% necrosis were selected. The tissues were subjected to nucleic acids extraction using Allprep DNA/RNA/miRNA Universal Kit (Qiagen, Hilden, Germany) according to the manufacturer’s recommendations. The integrity of DNA and RNA was assessed using agarose gel electrophoresis and RNA 6000 Kit (Agilent Technologies, Santa Clara, CA, USA). The quantity and purity for both DNA and RNA were assessed using Nanodrop 2000c Spectrometer (Thermo Fisher Scientific, Waltham, MA, USA).

### 2.2. Bisulfite Conversion

Five hundred nanograms (500 ng) of DNA was chemically modified to convert all unmethylated cytosine to uracil by the EZ DNA methylation–Gold kit (Zymo Research, Irvine, CA, USA) according to the manufacturer’s protocol. The effectiveness of bisulfite conversion was determined using Universal Methylated DNA Standard & Control Primers (Zymo Research, Irvine, CA, USA) according to the manufacturer’s protocol.

### 2.3. Methylation Microarray

The Infinium DNA MethylationEPIC assay was performed on 12 patients according to the manufacturer’s specifications and the beadchips were scanned using iScan (Illumina, Inc., San Diego, CA, USA). The Illumina Infinium DNA MethylationEPIC assay examines the DNA methylation status of >850,000 CpG dinucleotides distributed over the whole genome, including >220,000 open chromatins.

### 2.4. Microarray Data Analysis

The raw idat files obtained from methylation microarray were analysed using GenomeStudio V1.9.0 and ChAMP Bioconductor packages [18]. Filters were applied to all datasets where CpG sites with a detection of *p*-values greater than 0.01 in one or more samples were excluded from further analysis. Additionally, probes on sex chromosomes were also removed. The raw intensities were SWAN-normalized to reduce the technical biases inherent in the probe design before statistical analysis [19]. In addition, to remove variation related to the beadchip and/or position, ComBat normalization was implemented [20]. Once normalization has been performed, β-values were extracted and subjected to further statistical analysis. Heatmap was generated using the online tool Morpheus (https://software.broadinstitute.org/morpheus/, Cambridge, MA, USA).

### 2.5. Gene Expression Analysis

The expression of *OPLAH* was validated in 51 CRC patients using Thunderbird SYBR qPCR Mix (Toyobo Co. Ltd., Osaka, Japan) on the CFX96TM Real-Time PCR Detection System (Bio-Rad, Hercules, CA, USA). GAPDH served as the reference gene. All primers were obtained from Integrated DNA Technologies (Hs.PT.58.22507981 for OPLAH and Hs.PT.39a.22214836 for GAPDH). Fold change of expression was calculated using the 2 (-DeltaDeltaC(T)) method [21].

### 2.6. Bisulfite Sequencing Validation

The methylation of *OPLAH* cg26256223 was validated in an additional 27 CRC patients. Due to limited DNA available, we were unable to perform the validation in the same samples subjected to microarray. Methyl Primer Express Software v1.0 (Thermo Scientific, Massachusetts, USA) was used for primer design and the forward primer’s sequence is 5′ CSRTTTYGGGGTTAAATTAAA 3′ while the reverse sequence is 5′ CCCCTAATCTCTCTAAACTCCTC 3′. PCR amplification was performed using 30 ng/µL bisulfite-converted DNA and HotStar Taq Master Mix (Qiagen, Hilden, Germany). The amplified PCR products were purified, cloned and sequenced. The fasta files were analysed using Bioedit v7.0.5.3 [22] and BISMA [23].

### 2.7. In Silico Validation of OPLAH Methylation

The methylation of OPLAH cg26256223 was validated based on in silico analysis of TCGA COAD dataset [3] using Wanderer [24].

### 2.8. Statistical Analysis

Differentially methylated CpG sites were determined using t statistics from the limma Bioconductor package [25,26]. We further used the filtering characteristic of adjusted *p*-value < 0.05 and Δβ of |0.3| to identify significant differentially methylated open chromatins. To verify the accuracy and specificity of the differentially methylated probes, the discriminative performance of the probes was assessed by receiver operating characteristic (ROC) curves, and the area under the ROC curve (AUC), sensitivity, and specificity at the optimal cut-offs were calculated. ROC analysis was performed using GraphPad Prism V8 (GraphPad Software, Inc., San Diego, CA, USA).

## 3. Results

### 3.1. Demography

The majority (n = 38) of the patients are between 60 to 70 years old, while the remaining (n = 13) were above 70. Thirty-five patients were at stage T3, followed by stage T2 (n = 6), T4 (n = 6) and stage T1 (n = 4). There was a balanced distribution of male and female patients. All of the tumours were adenocarcinoma and well-differentiated.

### 3.2. The Output from Infinium DNA MethylationEPIC Assay

The genome wide CpG methylation profiles of 12 pairs of CRC tissue and adjacent normal mucosa were generated using the Infinium DNA MethylationEPIC assay. Methylation level at each locus was assessed using β-values generated by the Illumina GenomeStudio software, which was based on the intensity of the methylated and unmethylated probes. Prior to downstream analysis, the Detection Score filter was used, leaving only loci with significantly higher mean signal intensities from multiple probes for a given CpG locus than those of the negative control in the same set of chip data (detection *p*-value < 0.05). The number of CpG loci with detection *p*-value < 0.05 range from 863,728 to 865,500, and the call rates from 99.6 to 99.8. Next, the controls information was retrieved from the built-in Control Dashboard, and all of our samples passed the quality controls which include Staining Controls, Extension Controls, Hybdridisation Controls, Target Removal Controls, Bisulfite Conversion Controls, Specificity Controls, Non-Polymorphic Controls, Negative Controls and Restoration Control. No samples were identified as outliers, suggesting a uniformed amplification and hybridization conditions for all samples. Further hierarchical clustering analysis of the raw data successfully group the samples according to cancer or normal group. Additionally, the raw data of microarray methylation can be obtained in GEO under accession GSE149282.

### 3.3. Locations of Differentially Methylated Open Chromatins

**Figure 1:**
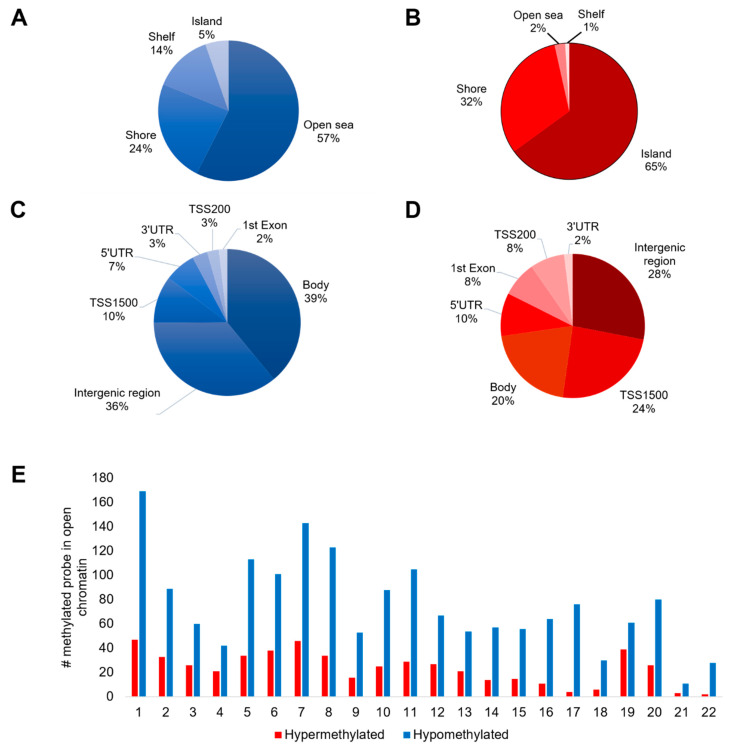
Differentially methylated open chromatins in CRC. (**A**) Distribution of hypomethylated open chromatins with respect to CGI features. (**B**) Distribution of hypermethylated open chromatins with respect to CGI features. (**C**) Distribution of hypomethylated open chromatins with respect to genomic regions. (**D**) Distribution of hypermethylated open chromatins with respect to genomic regions. (**E**) Chromosome-wise distribution of hypo- and hypermethylated open chromatins.

We compared the differential methylation status of 12 CRC tissue samples with the 12 adjacent cancer-free colonic tissue samples. Only differentially methylated regions with absolute delta β-values of at least 0.3 at adjusted *p*-value < 0.05 were reported. From the list of differentially methylated probes, we further filtered for those located in the open chromatin regions. Here, we found that the CRC-associated differentially methylated probes are located at 2187 open chromatin regions. The genomic and gene-related regions of the significant differentially methylated open chromatins were distributed in a different way. Generally, 517 probes were hypermethylated compared to 1670 probes sites that were hypomethylated. Figure 1A shows that the largest portion of hypomethylated sites (57%) were in the opensea and subsequently decreased in other categories (shore 24%, shelf 14% and island 5%). In contrast, more than half (65%) of the significantly hypermethylated open chromatins were in the island, followed by the shore (32%), opensea (2%) and shelf (1%) (Figure 1B). Meanwhile, Figure 1C shows that majority of the significantly hypomethylated loci were in the body (39%), closely followed by intergenic regions (36%), TSS1500 (10%), 5′ UTR (7%), 3′ UTR (3%), TSS200 (3%) and 1st exon (2%). However, more than a quarter (28%) of the significant hypermethylated loci were not associated with any genes, while the rest were mainly located in TSS1500 (24%), gene body (20%) and, to a lesser extent, in other gene categories including 5′ UTR, 1st exon, TSS200 and 3′ UTR (Figure 1D). Chromosome-wise, chromosome 1 has the highest number of hyper- and hypomethylated loci in open chromatin (47 and 169, respectively), followed by chromosome 7 (46 and 143, respectively) (Figure 1E).

### 3.4. Differentially Methylated Open Chromatins

**Figure 2:**
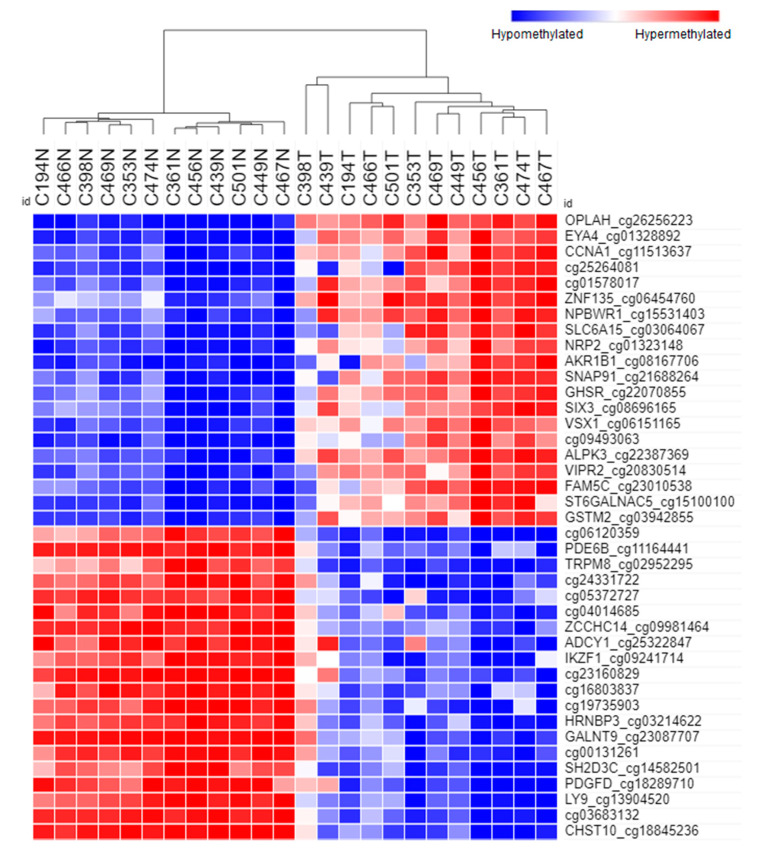
The hierarchical clustering and heatmap represent 40 significant methylation loci for open chromatin regions (20 hypermethylated and 20 hypomethylated). The dendrogram shows that all the samples are distinguished according to cancer (T) and normal (N) groups. The red boxes refer to the hypermethylated loci while the blue boxes denote the hypomethylated loci.

**Figure 3:**
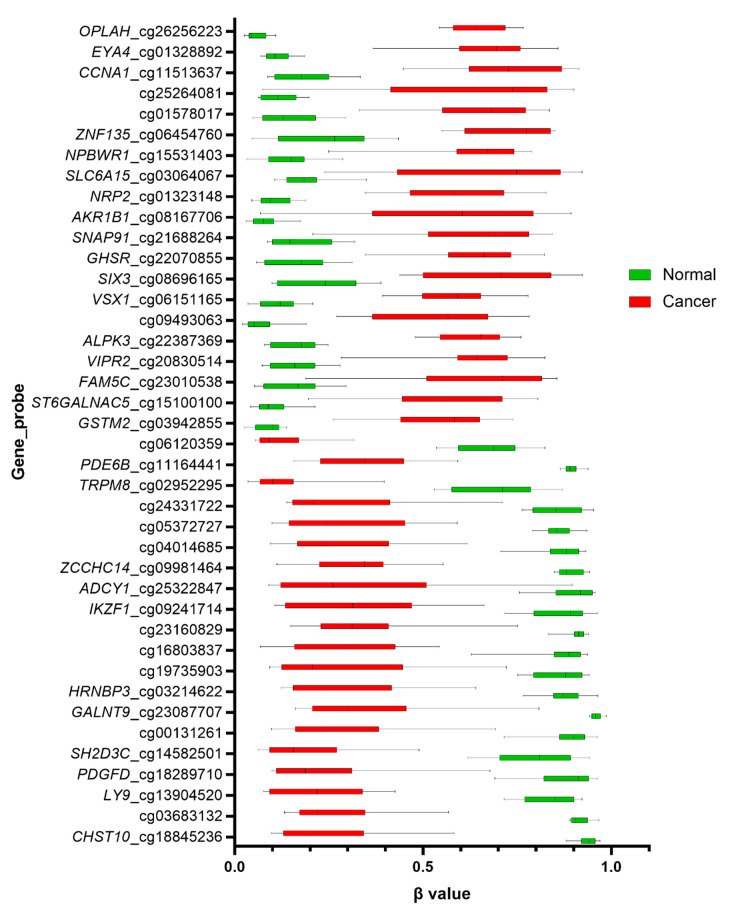
Box plot illustrating the comparison of β values for open chromatin probes in cancerous and normal tissues. The red boxes represent the methylation level in the cancer tissues, while the green boxes represent the methylation level in normal tissue. The top and bottom hinges of the box represent the highest and lowest values, while the thick horizontal lines within the box represent the mean β values.

Significant methylation differences for open chromatin regions were generated and illustrated through the heat map in Figure 2. Unsupervised hierarchical clustering based on Euclidean distance resulted in a distinct separation between cancer and normal tissues (Figure 2). The 40 topmost significant differentially methylated probes in open chromatins are illustrated in Table 1 while Figure 3 describes the box plot illustrating the comparison of β values for open chromatin probes in cancerous and normal tissues. The five hypermethylated probes with the highest Δβ values were *OPLAH* cg26256223 (Δβ = 0.603, adjusted *p*-value = 3.61 × 10^13^), *EYA4* cg01328892 (Δβ = 0.556, adjusted *p*-value = 1.58 × 10^9^), *CCNA1* cg11513637 (Δβ = 0.549, adjusted *p*-value = 1.54 × 10^8^), cg25264081 (Δβ = 0.517, adjusted *p*-value = 4.22 × 10^5^) and cg01578017 (Δβ = 0.511, adjusted *p*-value = 2.65 × 10^7^). On the other hand, the five hypomethylated probes with the highest reduction of methylation were *CHST10* cg18845236 (Δβ = −0.679, adjusted *p*-value = 3.66 × 10^9^), cg03683132 (Δβ = −0.639, adjusted *p*-value = 8.59 × 10^10^), *LY9* cg13904520 (Δβ = −0.622, adjusted *p*-value = 6.73 × 10^10^), *PDGFD* cg18289710 (Δβ = −0.615, adjusted *p*-value = 7.79 × 10^7^) and *SH2D3C* cg14582501 (Δβ = −0.610, adjusted *p*-value = 3.43 × 10^9^).

Of 2187 differentially methylated CpGs identified in the probe-level test, 1443 (66%) were mapped to 1025 genes. *TNXB* gene has the highest number of differentially methylated open chromatins (n = 19), followed by *HRNBP3* (n = 14), in which all loci were hypomethylated. Eighteen loci were in the *TNXB* gene body and one locus was located in the 5′ UTR. On the contrary, 5′ UTR is the most common hypomethylated sites in *HRNBP3*, with only one locus in the gene body. Other than these 2 genes, the majority (n = 804; 78%) of the genes had only one differentially methylated site.

### 3.5. Receiver Operating Characteristics (ROC) Curve Analysis

**Figure 4:**
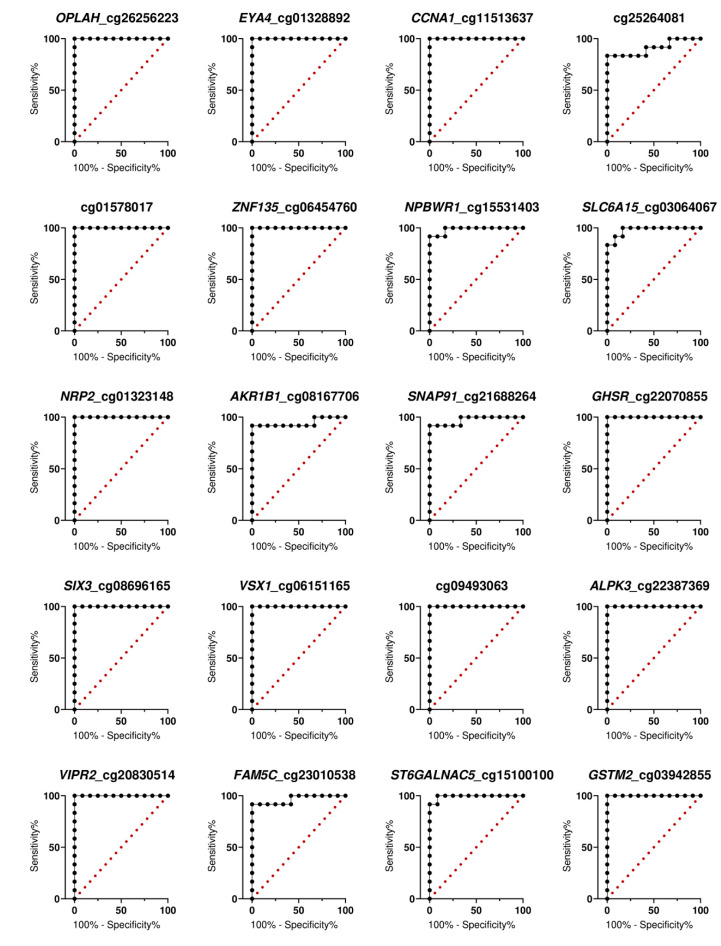
ROC curve-based evaluation of the diagnostic accuracy of hypermethylated open chromatin methylation markers.

**Figure 5:**
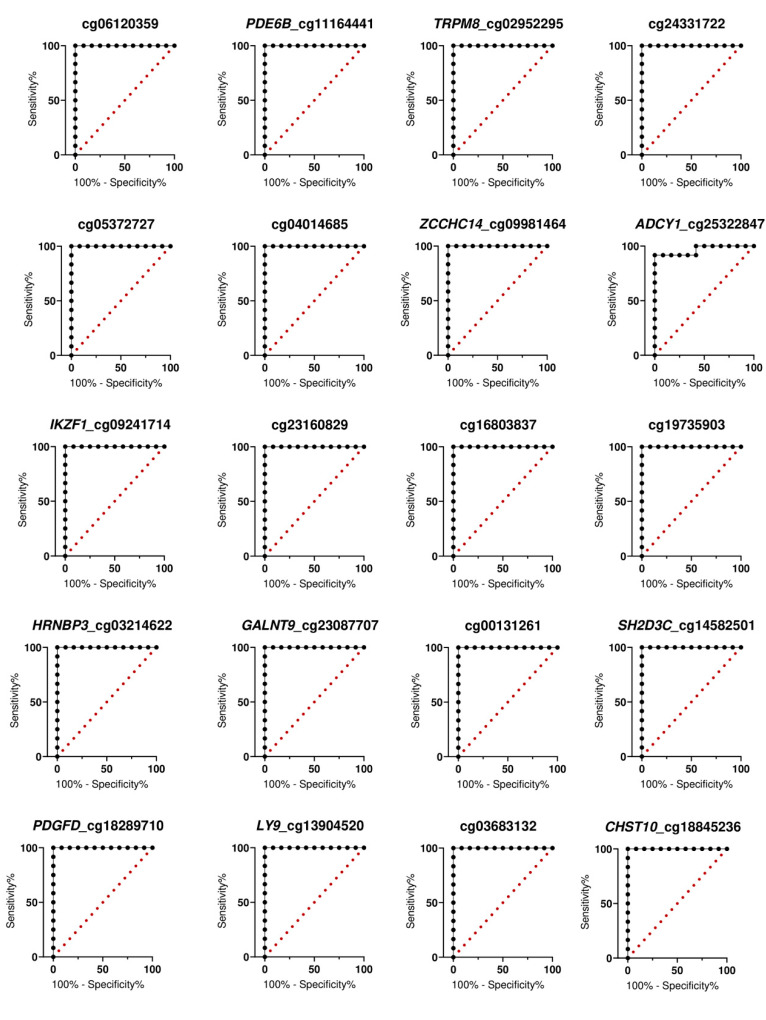
ROC curve-based evaluation of the diagnostic accuracy of hypomethylated open chromatin methylation markers.

Finally, the specificity and sensitivity of the methylation levels were evaluated using receiver operator curve (ROC) analysis. The methylation levels at all 40 topmost CpG sites significantly distinguished the CRCs from the normal colonic tissues (*p*-value < 0.001) (Table 2). The highest discriminative accuracy was demonstrated by 32 loci (80%), including *OPLAH* cg26256223, *EYA4* cg01328892, *CCNA1* cg11513637, cg01578017, *ZNF135* cg06454760, among others (AUC = 1, confident interval = 1–1). Other candidate markers also reached particularly high diagnostic accuracy (AUC = 0.9097–1, *p*-value < 0.001; Table 2; Figure 4 and Figure 5).

### 3.6. Validation of OPLAH cg26256223 Methylation and Global Expression in CRC

The methylation pattern of *OPLAH* across 27 CRC samples using bisulfite sequencing was in concordance with the methylation profiling. The validation showed *OPLAH* exhibit average 51.9% of hypermethylation in CRCs in which the methylation percentage is close to the methylation value in microarray.

**Figure 6:**
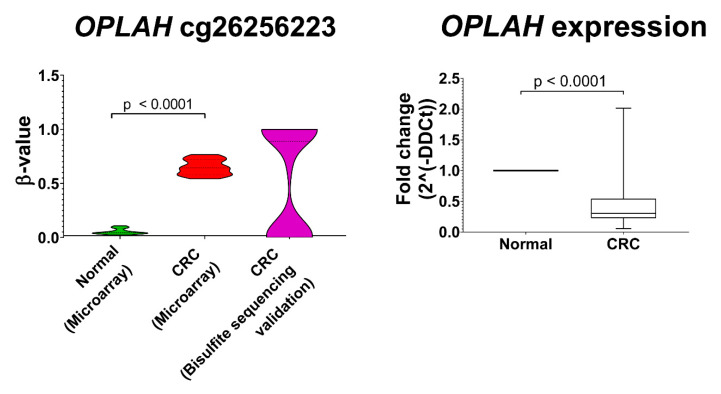
Significant *OPLAH* downregulation was observed in the cancer tissues (CRC) compared to the normal colon. *OPLAH* cg26256223 hypermethylation was also validated in our validation samples.

In order to investigate whether *OPLAH* hypermethylation, in particular the locus cg26256223, resulted in changes in gene expression, a qPCR analysis was performed. In line with our hypothesis, the expression of *OPLAH* was significantly reduced in CRC compared to normal tissues (*p* < 0.0001, fold change = −2.167) (Figure 6).

### 3.7. In Silico Validation Using TCGA Dataset

**Figure 7:**
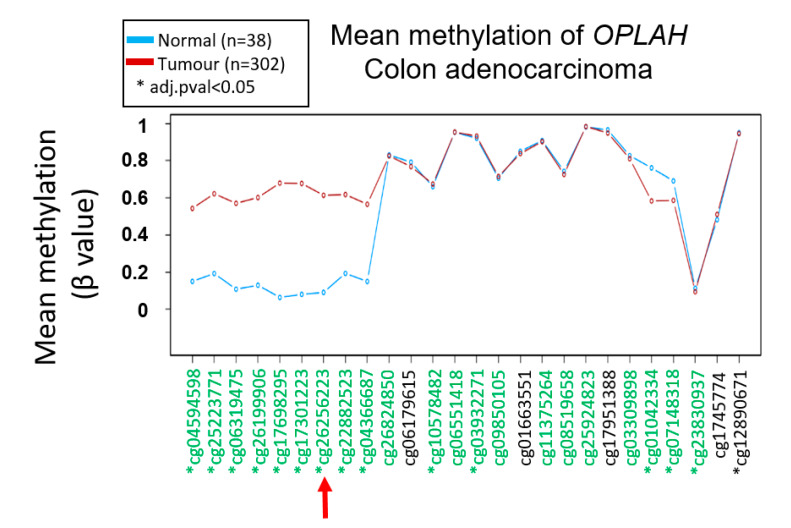
In silico validation of *OPLAH* methylation in the TCGA COAD dataset. The red arrow indicates the specific hypermethylated locus identified in our study, which is also found to be significantly hypermethylated in the tumour compared to the normal colon.

Additionally, we also performed in silico validation of *OPLAH* methylation to determine whether our finding agrees with the study by TCGA. Fifteen CpG loci in *OPLAH* were found to be significantly hypermethylated in 302 CRCs versus 38 normal tissues, including our locus of interest (Figure 7).

## 4. Discussion

An array-based analysis is a simple, practical, and cost-effective tool for genome-wide DNA methylation screening. The latest DNA methylation microarray, Infinium DNA MethylationEPIC, provides substantively increased genomic coverage than previous studies, permitting the identification of novel methylated CpG sites that have not been previously discovered. In this study, we focused on the genome-wide methylation patterns of open chromatins among CRC patients. Data analysis showed that there were more hypomethylated loci than the hypermethylated counterpart, an observation supported by several other studies [10,14,16]. Our finding is further supported by a review [27] which postulates that hypomethylated genes include those involved in nucleosome and chromatin formation, in which the latter is the focus of our study. We also noticed that the hypomethylated sites were predominant at the opensea, while hypermethylation was more common on the island. Since there is no published literature that specifically focuses on the open chromatins using Infinium DNA MethylationEPIC in CRC, we are unable to verify this finding. Nevertheless, from the overall perspectives, our finding is in line with Baharudin et al. [10] and Naumov et al. [28]. Openseas are the regions located more than 4 kilobases from CpG islands, where hypomethylation was the prevalent form of aberrant methylation [29]. CpG islands, on the other hand, are short stretches of CpG-rich sequence which often aberrantly hypermethylated in cancers [30].

The majority of the significantly hypomethylated loci in this study were in the body, while more than a quarter (28%) of the significant hypermethylated loci were in the intergenic regions hence not associated with any genes. Our findings are in disagreement with another study [28] which reported majority (30.6%) the significantly hypomethylated CpG sites were in intergenic regions, while the largest portion of hypermethylated CpGs (25.9%) were in the 1st exon. DNA methylation in the intergenic regions was shown to regulate microRNA expression [31] and stabilizes the genome [32]. Furthermore, Hanley and colleagues discovered that intergenic methylated loci are enriched for transcription factor binding sites, particularly the AP-1 transcription factor family that regulates important cellular functions including apoptosis, proliferation, and differentiation [33]. The only published study on CRC based on Infinium DNA MethylationEPIC [16] did not describe the analysis at the gene-region level, therefore we were unable to compare. Nevertheless, gene body hypomethylation has been reported in cancer and was shown to be linked with reduced transcription activities compared to normal cells [34,35,36].

We identified 2187 differentially methylated sites, of which 1443 (66%) were mapped to 1025 genes. The involvement of several of those genes in CRC has been previously exposed, although the contribution of other genes in colorectal carcinogenesis is the subject for further research. It is noteworthy to mention that profiles of *OPLAH* cg26256223, *EYA4* cg01328892 and *CCNA1* cg11513637, cg25264081 hypermethylation in this study were similar to the patterns reported elsewhere [28,37,38]. Luo and colleagues [37] compared methylomes of the normal colon mucosa, tubular adenomas, as well as CRC and reported hypermethylation of *EYA4* cg01328892 in CRC and adenoma compared to normal. There is no evidence supporting the hypermethylation of cg01578017 in CRC. Conversely, *CHST10* hypermethylation in CRC compared to normal has been reported in several studies [39,40], a disagreement with our finding in which *CHST10* cg18845236 was found to be hypomethylated. It is unclear which specific loci those studies referred to. Additionally, hypomethylation status of cg03683132, *LY9* cg13904520, *PDGFD* cg18289710 and *SH2D3C* cg14582501 in CRC has not been reported.

*OPLAH* (human 5-oxoprolinase) is a gene involved in ATP-hydrolysis located on chromosome 8q24.3 [41]. Our preliminary finding suggested that the differentially methylated *OPLAH* cg26256223 had a significant effect on gene regulation, suggesting a possible contribution to CRC through transcriptome alteration. To date, *OPLAH* cg26256223 was also reportedly hypermethylated in CRCs [28,38]. Despite limited literature pertaining to *OPLAH* methylation in CRC, there are already several patents field for its application in cancer detection (https://link.lens.org/id5DmKPvsRe, Canberra, Australia). Forty-six patents were granted, with another 244 patents filed. The biggest applicant is the Mayo Foundation for Medical Education & Research, with a total of five patents granted and 17 patents applied. One of their patents, US 10,370,726 B2, is a patent granted for the use of *OPLAH*, among other genes, to detect CRC in individuals younger or older than 50 years old or in Lynch Syndrome patients [42]. Evidently, the capacity of *OPLAH* for CRC diagnostic has been proven. Further studies on its role in the prognosis of disease, the response to treatment and the exploration of its druggable potential are worth investigating.

Barrow and colleagues [43] performed the epigenome-wide analysis of DNA methylation in CRC with different smoking statuses; 36 never smokers, 47 former smokers and 13 active smokers, as well as adjacent mucosa from 49 never smokers, 64 former smokers and 18 active smokers. The authors reported significant hypomethylation of four loci associated with the *TNXB* gene in tissue from active smokers. In our study, we identified 19 hypomethylated *TNXB* loci in CRC compared to the normal colon; however, the association with smoking status could not be established due to the lack of information. *TNXB* (tenascin XB) gene encodes a tenascin, which exhibits an anti-adhesive effect [44]. It was first implicated in Ehlers-Danlos syndrome [45], but its role in malignancy has also been established in several cancers including nasopharyngeal [46] and mesothelioma [47], most possibly by promoting the epithelial-to-mesenchymal transition (EMT) to activating latent transforming growth factor-β [48]. More recently, *TNXB* is denoted as one of the triple-evidenced genes, displaying superior predictive ability in cancer diagnosis and prognosis [49].

*HRNBP3* (RNA binding fox-1 homolog 3) gene encodes for an RNA-binding protein and regulates the alternative splicing of pre-mRNA. A meta-analysis revealed that the *HRNBP3* gene was one of the most commonly hypomethylated genes in hepatocellular carcinoma [50,51]. In contrast with our finding, Hua et al. [38] reported modest hypermethylation of *HRNBP3* in one locus from The Cancer Genome Atlas’ rectal carcinoma study. Another study pertaining to this gene in CRC is still lacking, therefore we are the first to report *HRNBP3* hypomethylation in CRC, in which 14 loci in this gene were hypomethylated. In line with its function as RNA binding protein, majority of the hypomethylation was in the 5′ UTR, a region important for regulation of translation [52].

## 5. Conclusions

Our work gives a detailed assessment of the DNA methylation pattern of open chromatins and revealed epigenetically regulated candidate genes in CRC carcinogenesis. Specifically, our results provide the first evidence of *HRNBP3*, cg03683132, *LY9* cg13904520, *PDGFD* cg18289710 and *SH2D3C* cg14582501 hypomethylation in CRC. This is the first insight on the open chromatins methylation profile in Malaysian CRC patients. The new knowledge from this study can be utilized to further increase our understanding of CRC methylomics, particularly on the open chromatins. To minimize the effect of confounding factors, methylome studies should be performed in cancer and adjacent normal tissues that have been collected from the same individual, as demonstrated in this study, instead of collecting cancer and normal tissues from a different individual.

However, our study is not without limitation. Although our sample size is small and lack of functional studies, the hypo- and hypermethylation of the genes reported in this study are relevant to carcinogenesis as reported in several studies. In future, the association with survival and other clinicopathological data is warranted. With regards to the heterogeneity of bulk tissue methylomics, single-cell epigenomic shall be performed to obtain higher resolution, cancer-specific methylation changes in order to better understand this cancer at the cellular level. In conclusion, the prognostic and diagnostic roles of the differentially methylated open chromatins warrant future investigations.

## Figures and Tables

**Table 1:** Forty topmost significant differentially methylated probes in open chromatins.

Gene_Probe	Delta Beta (Δβ)	Adjusted *p*-Value	Region	Chromosome
*OPLAH_*cg26256223	0.603	3.61 × 10^13^	*Body-island*	8
*EYA4_*cg01328892	0.556	1.58 × 10^9^	*TSS200-island*	6
*CCNA1_*cg11513637	0.549	1.54 × 10^8^	*TSS1500-shore*	13
cg25264081	0.517	4.22 × 10^5^	*IGR-island*	13
cg01578017	0.511	2.65 × 10^7^	*IGR-shore*	7
*ZNF135_*cg06454760	0.503	5.91 × 10^8^	*TSS200-island*	19
*NPBWR1_*cg15531403	0.497	5.53 × 10^8^	*TSS1500-island*	8
*SLC6A15_*cg03064067	0.491	1.60 × 10^5^	*TSS1500-shore*	12
*NRP2_*cg01323148	0.489	2.79 × 10^8^	*Body-island*	2
*AKR1B1_*cg08167706	0.489	5.38 × 10^5^	*TSS1500-shore*	7
*SNAP91_*cg21688264	0.487	2.57 × 10^6^	*Body-island*	6
*GHSR_*cg22070855	0.478	7.10 × 10^8^	*TSS1500-island*	3
*SIX3_*cg08696165	0.477	1.55 × 10^6^	*3′UTR-shore*	2
*VSX1_*cg06151165	0.475	2.04 × 10^9^	*Body-island*	20
cg09493063	0.473	3.61 × 10^7^	*IGR-island*	7
*ALPK3_*cg22387369	0.471	5.30 × 10^10^	*1stExon-shore*	15
*VIPR2_*cg20830514	0.468	6.44 × 10^7^	*Body-island*	7
*FAM5C_*cg23010538	0.467	2.01 × 10^5^	*TSS1500-opensea*	1
*ST6GALNAC5_*cg15100100	0.466	6.83 × 10^7^	*TSS200-shore*	1
*GSTM2_*cg03942855	0.464	3.38 × 10^8^	*Body-island*	1
cg06120359	−0.549	8.20 × 10^10^	*IGR-opensea*	8
*PDE6B_*cg11164441	−0.549	1.42 × 10^9^	*Body-shore*	4
*TRPM8_*cg02952295	−0.555	1.28 × 10^8^	*Body-opensea*	2
cg24331722	−0.556	3.36 × 10^7^	*IGR-opensea*	8
cg05372727	−0.560	5.43 × 10^8^	*IGR-opensea*	13
cg04014685	−0.562	8.56 × 10^8^	*IGR-opensea*	3
*ZCCHC14_*cg09981464	−0.563	1.31 × 10^9^	*3′UTR-island*	16
*ADCY1_*cg25322847	−0.563	2.21 × 10^5^	*Body-shelf*	7
*IKZF1_*cg09241714	−0.564	1.38 × 10^7^	*Body-opensea*	7
cg23160829	−0.565	6.11 × 10^8^	*IGR-opensea*	6
cg16803837	−0.572	5.57 × 10^8^	*IGR-opensea*	7
cg19735903	−0.573	2.50 × 10^7^	*IGR-opensea*	13
*HRNBP3_*cg03214622	−0.585	1.09 × 10^8^	*5′UTR-opensea*	17
*GALNT9_*cg23087707	−0.590	1.81 × 10^7^	*Body-shore*	12
cg00131261	−0.596	3.27 × 10^8^	*IGR-opensea*	8
*SH2D3C_*cg14582501	−0.610	3.43 × 10^9^	*Body-shelf*	9
*PDGFD_*cg18289710	−0.615	7.79 × 10^7^	*Body-opensea*	11
*LY9_*cg13904520	−0.622	6.73 × 10^10^	*Body-opensea*	1
cg03683132	−0.639	8.59 × 10^10^	*IGR-opensea*	12
*CHST10_*cg18845236	−0.679	3.66 × 10^9^	*Body-opensea*	2

IGR—intergenic regions; UTR—untranslated regions, TSS—transcription start sites.

**Table 2:** Receiver operating characteristics (ROC) curve analysis of the differentially methylated probes in open chromatins.

Gene	Probe	AUC	95% Confident Interval	*p* Value
*OPLAH*	cg26256223	1.0000	1.000–1.000	<0.0001
*EYA4*	cg01328892	1.0000	1.000–1.000	<0.0001
*CCNA1*	cg11513637	1.0000	1.000–1.000	<0.0001
	cg25264081	0.9097	0.7816–1.000	0.0007
	cg01578017	1.0000	1.000–1.000	<0.0001
*ZNF135*	cg06454760	1.0000	1.000–1.000	<0.0001
*NPBWR1*	cg15531403	0.9861	0.9505–1.000	<0.0001
*SLC6A15*	cg03064067	0.9792	0.9346–1.000	<0.0001
*NRP2*	cg01323148	1.0000	1.000–1.000	<0.0001
*AKR1B1*	cg08167706	0.9444	0.8357–1.000	0.0002
*SNAP91*	cg21688264	0.9722	0.9117–1.000	<0.0001
*GHSR*	cg22070855	1.0000	1.000–1.000	<0.0001
*SIX3*	cg08696165	1.0000	1.000–1.000	<0.0001
*VSX1*	cg06151165	1.0000	1.000–1.000	<0.0001
	cg09493063	1.0000	1.000–1.000	<0.0001
*ALPK3*	cg22387369	1.0000	1.000–1.000	<0.0001
*VIPR2*	cg20830514	1.0000	1.000–1.000	<0.0001
*FAM5C*	cg23010538	0.9653	0.8926–1.000	0.0001
*ST6GALNAC5*	cg15100100	0.9931	0.9708–1.000	<0.0001
*GSTM2*	cg03942855	1.0000	1.000–1.000	<0.0001
	cg06120359	1.0000	1.000–1.000	<0.0001
*PDE6B*	cg11164441	1.0000	1.000–1.000	<0.0001
*TRPM8*	cg02952295	1.0000	1.000–1.000	<0.0001
	cg24331722	1.0000	1.000–1.000	<0.0001
	cg05372727	1.0000	1.000–1.000	<0.0001
	cg04014685	1.0000	1.000–1.000	<0.0001
*ZCCHC14*	cg09981464	1.0000	1.000–1.000	<0.0001
*ADCY1*	cg25322847	0.9653	0.8926–1.000	0.0001
*IKZF1*	cg09241714	1.0000	1.000–1.000	<0.0001
	cg23160829	1.0000	1.000–1.000	<0.0001
	cg16803837	1.0000	1.000–1.000	<0.0001
	cg19735903	1.0000	1.000–1.000	<0.0001
*HRNBP3*	cg03214622	1.0000	1.000–1.000	<0.0001
*GALNT9*	cg23087707	1.0000	1.000–1.000	<0.0001
	cg00131261	1.0000	1.000–1.000	<0.0001
*SH2D3C*	cg14582501	1.0000	1.000–1.000	<0.0001
*PDGFD*	cg18289710	1.0000	1.000–1.000	<0.0001
*LY9*	cg13904520	1.0000	1.000–1.000	<0.0001
	cg03683132	1.0000	1.000–1.000	<0.0001
*CHST10*	cg18845236	1.0000	1.000–1.000	<0.0001
